# Standard radiographic assessments of distal radius fractures miss involvement of the distal radioulnar joint: a diagnostic study

**DOI:** 10.1007/s00402-021-03801-7

**Published:** 2021-02-08

**Authors:** Laura A. Hruby, Thomas Haider, Roberta Laggner, Claudia Gahleitner, Jochen Erhart, Walter Stoik, Stefan Hajdu, Gerhild Thalhammer

**Affiliations:** 1 Department of Orthopedics and Trauma Surgery, Medical University of Vienna, Spitalgasse 23, 1090 Vienna, Austria; 2 Department of Orthopedics and Traumatology, Hospital of the St. John of God Brothers Eisenstadt, Johannes von Gott-Platz 1, 7000 Eisenstadt, Austria

**Keywords:** Distal radius fracture, Fracture classification system, Interobserver reliability, Distal radioulnar joint

## Abstract

**Introduction:**

Distal radius fractures account for one-fifth of all fractures in the emergency department. Their classification based on standard radiographs is common practice although low inter-observer reliabilities and superiority of computer tomography (CT) scanning in evaluation of joint congruency have been reported.

**Materials and methods:**

We retrospectively analyzed 96 displaced distal radius fractures scheduled for open reduction and internal fixation using standard radiographic assessment. The radiographs were classified with the Arbeitsgemeinschaft für Osteosynthesefragen/Orthopaedic Trauma Association (AO/OTA), Fernandez and Frykman classifications by three observers and inter-rater reliabilities were calculated. Additional CT scanning was performed in all cases and the following parameters were assessed: radiocarpal joint involvement, fracture extent into the radial sigmoid notch, i.e. the distal radio-ulnar joint, comminution of the metaphysis, and concomitant ulnar styloid fracture. The CT scans were used as a reference standard to determine sensitivity and accuracy of standard radiographic assessment in evaluation of distal radius fractures.

**Results:**

The inter-rater agreement for the AO classification was 35.4%, 68.8% for the Fernandez and 38.5% for the Frykman classification. Fracture extension into the radiocarpal joint was present in 81 cases (84.4%). Sigmoid notch involvement was found in 81 fractures (84.4%). Involvement of both joints was present in 72 cases (75%). The sensitivity of standard radiographs regarding radiocarpal joint involvement was 93.8%. Considering involvement of the distal radio-ulnar joint the false-negative rate using standard radiographs was 61.7% and the test’s accuracy for sigmoid notch involvement was 45.8%.

**Conclusion:**

This study demonstrates that involvement of the sigmoid notch is frequently missed in standard radiographs. The presented data support the frequent use of CT imaging to allow the holistic illustration of a fracture’s complexion and to ensure optimal pre-operative planning.

## Background

Distal radius fractures (DRFs) are among the most frequent injuries of the upper extremity and account for one-fifth of all fractures seen in the emergency department [1, 2]. The acute management of DRFs depends on various clinical and radiological parameters [3]. For the initial assessment, two-plane radiographs remain the gold standard. However, precise evaluation in regards to intra-articular gaps and step-offs is limited with conventional radiography and computed tomography (CT) scans are known to allow for a better assessment of fracture complexion and extensions into articular surfaces [4].

Traumatic lesions of the distal radio-ulnar joint (DRUJ) occur in conjunction with fractures of the distal radius in a considerable number of cases and underappreciated injuries are a common cause of ulnar-sided wrist pain and limited range of motion [5–7]. Amongst the most common classification systems of distal radius fractures, however, only the Frykman classification [8] takes into account the involvement of the distal radio-ulnar joint. Few studies with limited patient numbers have compared distal radius fracture extensions using conventional radiographs and computer tomography (CT) [9–11], all of which found an underestimation of distal radio-ulnar joint involvement based on plain radiographs.

The purpose of this study was to compare fracture types and complexion of DRFs in standard radiographs and computed tomography (CT) scans. It was hypothesized that standard radiographic examinations underestimate involvement of both the radiocarpal and distal radioulnar joints.

## Methods

Between February 2017 and January 2020, radiographic and CT examinations in patients with distal radius fractures were obtained in 96 consecutive cases. All included patients were scheduled for surgical treatment, i.e. open reduction and internal fixation (ORIF) with volar plate osteosynthesis. The standardized radiographic assessment of the wrist consisted of posteroanterior and lateral projections. The CT scans (Siemens Somatom Edge plus, Siemens Healthineers, Germany) were performed in prone position with the arm stretched forward over the head with forearm and wrist in neutral position. Evaluation of CT scans included sagittal, coronal and axial reconstructions in the bone window. Standard radiographic projections were obtained at initial consultation after injury as well as after closed reduction with the forearm immobilized in a cast. The timing of the CT scan was not standardized and ranged from immediately post reduction up to fifteen days after injury with the forearm immobilized in a cast.

Three observers independently evaluated anonymized radiographs and classified the fractures according to the AO/OTA [12], Fernandez [13] and Frykman [8] classification systems. The three observers were an orthopedic trauma surgery resident at the beginning of training, one at the end of training, as well as an orthopedic trauma surgery attending with specialization in hand surgery with over 15 years of clinical experience. No consensus was negotiated throughout the assessment of conventional radiographs. During evaluation of the digital images, schematic drawings of the respective classification systems were available on site for each observer.

Since additional CT scans were shown not to increase reliability of the mentioned classification systems [9, 10], the CT scans were evaluated by all 3 investigators holding consensus meetings. Each fracture was evaluated blinded in a randomized order. Axial, coronal, and sagittal reconstructions through both articular surfaces were evaluated in all cases. The following parameters were discussed and assessed: (1) radiocarpal joint involvement, (2) fracture extent into the radial sigmoid notch, i.e. the distal radio-ulnar joint, (3) comminution of the metaphysis, and (4) concomitant ulnar styloid fracture. To facilitate comparability of the results in terms of absolute and relative frequencies, the CT scans were also classified using the three mentioned classification systems. Since it was hypothesized that the experienced hand-specialized trauma surgeon would most accurately identify the true fracture morphology on plain radiographs, the CT images were compared to this observer’s results.

To elucidate differences in correct fracture assessment related to clinical experience, results between observer 1, i.e. the orthopedic trauma surgery resident at the end of training, and observer 2, i.e. the orthopedic trauma surgery attending with specialization in hand surgery with over 15 years of clinical experience, were directly compared.

### Statistics

Data were analysed using the statistical program R (version 3.6.1). Absolute and relative frequencies of all fractures are given. To describe inter-rater reliability of the respective fracture classification systems agreements are presented as percentage values. Sensitivity, false-negative rate and accuracy of radiographic assessments were calculated using CT scans as the true reference.

## Results

### Study participants

Ninety-six patients with DRF scheduled for ORIF with a mean age of 55 ± 11 years (range, 21–75) were included. Seventy-seven patients (80%) were female.

### Fracture types comparing standard radiographs and CT scans

Table 1 gives absolute values and relative frequencies of the classified fractures comparing conventional radiographs and computer tomography scans. In 15 cases (15.6%), extra-articular fractures were diagnosed with the CT, while the radiocarpal joint alone was affected in 9 patients (9.4%). Fracture extensions into the sigmoid notch occurred in 81 cases (84.4%), while this was combined with radiocarpal joint involvement in 72 cases (75%). In total, the radiocarpal joint was affected in 81 cases (84.4%).

**Table 1:** Absolute frequencies of the fracture distributions comparing standard radiographs (R) and computer tomography scans (CT)

	R	CT
AO		
A1	0	0
A2	2	0
A3	14	15
B1	0	0
B2	0	0
B3	4	1
C1	19	1
C2	43	35
C3	14	44
Fernandez		
I	16	15
II	4	1
III	76	78
IV	0	1
V	0	1
Frykman		
I	3	1
II	11	5
III	20	5
IV	29	4
V	0	2
VI	3	7
VII	10	19
VIII	20	53

**Fig. 1:**
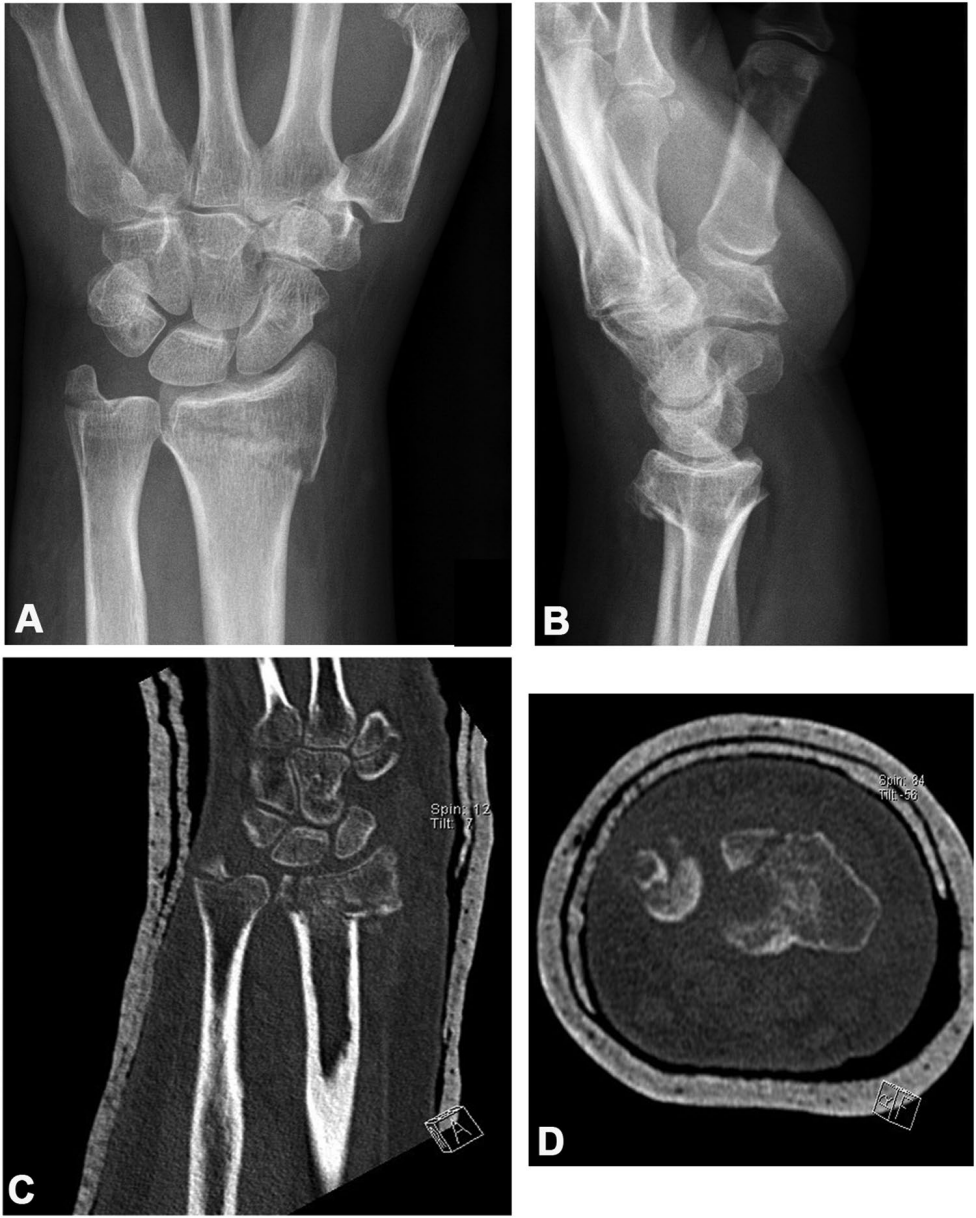
Distal radius fracture in a 63-year-old female patient. Considering the posteroanterior (**a**) and lateral (**b**) projections of plain film radiography, this fracture was classified as an extraarticular A2 fracture according to the AO/OTA classification. In the coronal (**c**) and axial (**d**) CT reconstructions, however, radiocarpal joint involvement was clearly identified

To outline differences in assessing radiocarpal joint involvement using plain radiographs and CT scans for a total of 96 cases, fourfold tables for two observers are shown (observer 1, i.e. an orthopedic trauma surgeon resident at the end of training; observer 2, i.e. a hand-specialized surgeon; see Table 2). While observer 1 correctly identified involvement of the radiocarpal joint using standard radiographs in 86.4% of all CT-verified articular fractures, the radiographic assessment’s sensitivity to recognize articular involvement increased to 93.8% for observer 2. Figure 1 shows a case, where radiocarpal joint involvement was missed in plain radiographs and apparent in the coronal and axial CT reconstructions.

**Table 2:** To compare radiocarpal joint involvement using radiographs (R) and computer tomography scans (CT) the AO/OTA classification (A, B, C) was used

Observer 1	CT (B + C)	CT (A)	
R (B + C)	70	5	75
R (A)	11	10	21
	81	15	96

Observer 2	CT (B + C)	CT (A)	
R (B + C)	76	4	80
R (A)	5	11	16
	81	15	96

Fourfold tables of the results for observers 1 and 2 are shown. “(A)” include extra-articular fractures, while “(B + C)” comprise intra-articular fractures

**Fig. 2:**
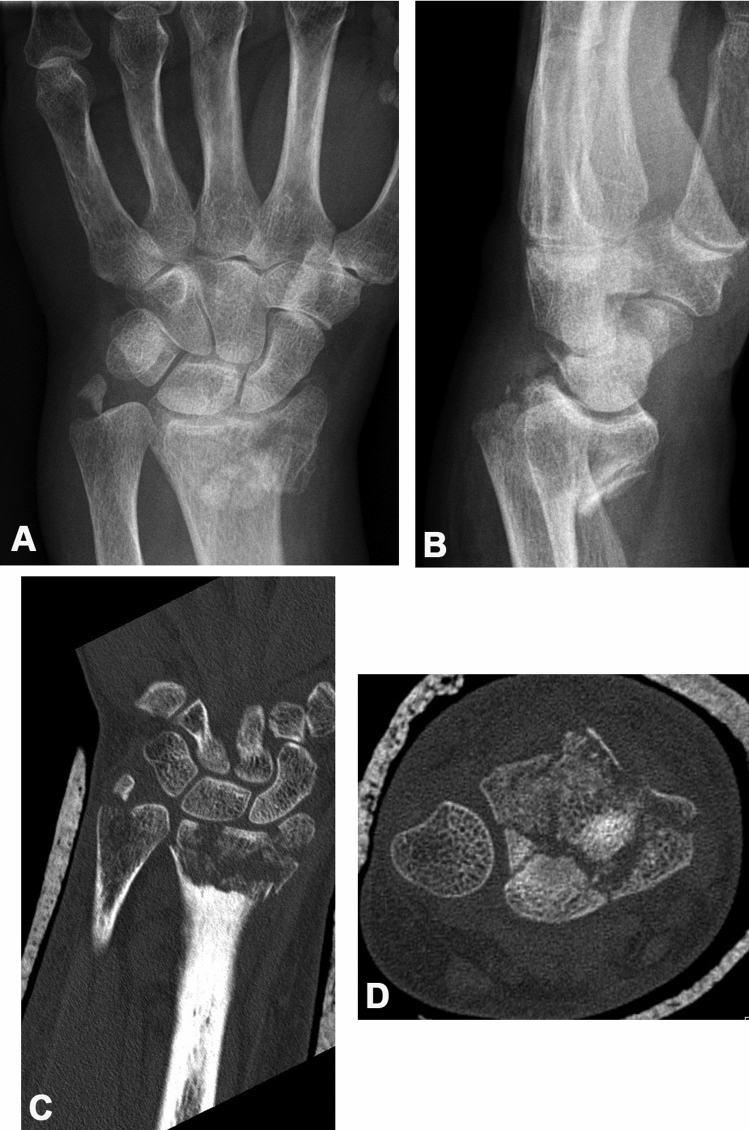
Distal radius fracture in a 61-year-old female patient. Using standard posteroanterior (**a**) and lateral (**b**) radiographs, this fracture was classified as a type IV fracture according to the Frykman classification. The coronal (**c**) and axial (**d**) CT reconstructions, however, clearly show fracture extension into the sigmoid notch

The distribution of recognized fracture extensions into the DRUJ comparing radiographs and CT scans was analyzed using the Frykman classification, since this is the only classification evaluating its involvement. Creating fourfold tables, the results for the same two observers were compared (see Table 3). The sensitivity of the standard radiographic assessment to identify DRUJ involvement was 33.3% for observer 1 and 38.3% for observer 2. When CT scans were considered as the reference, the false-negative rate of standard radiographs for DRUJ involvement therefore was 66.6% and 61.7%, respectively, and the test’s accuracy was 39.6% and 45.8%, respectively. Figure 2 shows a case, where extension of the fracture into the sigmoid notch was not seen on standard radiographs, however, was obvious when coronal and axial CT reconstructions were evaluated.

**Table 3:** To compare distal radio-ulnar joint (DRUJ) involvement using radiographs (R) and computer tomography scans (CT), the Frykman classification (I–VIII) was used

Observer 1	CT (V–VIII)	CT (I–IV)	
R (V–VIII)	27	4	31
R (I–IV)	54	11	65
	81	15	96

Observer 2	CT (V–VIII)	CT (I–IV)	
R (V–VIII)	31	2	33
R (I–IV)	50	13	63
	81	15	96

Fourfold tables of the results for observers 1 and 2 are shown. “(I–IV)” comprise fractures not involving the DRUJ, whil (V–VIII) include fractures with DRUJ involvement

Regarding comminution of the metaphysis, underestimation of a multi-fragmentary fracture occurred in 23 of 96 cases (24.0%) using standard radiographs. Osseous avulsion of the ulnar styloid process was underappreciated in 12 of 96 cases (12.5%).

### Reliability of the AO/OTA, Fernandez and Frykman classifications using standard radiographs

The agreement for all observers was 35.4% for the AO/OTA classification, 68.8% for the Fernandez classification, and 38.5% for the Frykman classification. For clustering fracture types only into A, B and C types, absolute agreement increased to 69.8% for the AO/OTA classification. Regarding the Frykman classification, in 56.2% of the cases agreement among all three observers was obtained regarding distal radio-ulnar joint involvement.

## Discussion

Conventional radiographs in two planes remain the gold standard in assessing distal radius fractures in the emergency department. Using only plain radiographs, however, it is hardly possible to recognize the details of a complex fracture as multiple bone fragments overlap in one plane and the precise evaluation of gaps and step-offs in joint surfaces is inaccurate [4]. Also, DRFs in the acute setting are often associated with imperfect projection planes due to pain and concomitant loss of active and passive wrist motion. The superiority of CT scanning in evaluation of distal radius fractures has been shown by a number of authors [4, 14–16]. Still, however, its application in clinical decision-making, i.e. conservative versus operative treatment, as well as in pre-operative planning is not universally accepted [17]. The prerequisite of a useful classification system lies in the organization of clinical information in a manner that is consistent from one observation to the next [18]. However, the commonly applied systems, i.e. the AO/OTA, Frykman and Fernandez classifications, fail to fulfill these criteria. Poor inter- and intra-observer reliabilities have been shown for all mentioned classifications in various studies [10, 11, 17, 19, 20]. The presented results of percent agreement amongst the three observers evaluating plain radiographs perfectly align with previously published results [9, 17–21]. As mentioned above, image quality of conventional radiographs greatly varies due to inaccurate projections and could possibly explain the low reliability of the common fracture classifications. Interestingly, several authors have shown that the inter-observer reliability of the various fracture classifications is not improved using additional computer tomography scanning [10]. It has to be noted that the original AO classification was originally designed for study purposes and neither of the mentioned classification systems were initially designed to evaluate fracture types with CT imaging. When axial, coronal and sagittal planes are incorporated in the fracture type evaluation a more accurate or rather “true” representation of the respective fracture may be recognized. Kleinlugtenbelt et al. [10] therefore concluded that the additional value of CT scanning over standard radiographic assessment is limited with regard to fracture classification reliability, but has significant implications for accurate evaluation of the fracture types. Flikkilä and colleagues [9], who also assessed the use of CT in regards to fracture classification using the AO/OTA system, came to a similar conclusion: CT is of minor value in improving the inter-observer reliability but offers much higher accuracy in evaluating joint involvement. In this study, CT images were analyzed in light of four distinct questions, i.e. radiocarpal and distal radio-ulnar joint involvements, metaphyseal comminution, and ulnar styloid fracture. Regarding radiocarpal joint involvement, the radiographic assessment’s sensitivity was 86.4% for an orthopaedic trauma surgeon at the end of training and 93.8% for a hand-specialized surgeon with over fifteen years of clinical experience. The sensitivity of conventional radiographic assessment regarding distal radio-ulnar joint involvement, however, dropped to 33.3% and 38.3%, respectively, when CT scans were considered as the true reference. Correct identification of fracture extensions into the DRUJ therefore did only negligibly improve with clinical experience and were missed in 61.7 to 66.6% of the cases. Generally, sigmoid notch involvement was found in 84.3% of all scanned fractures, which is similar or slightly higher than numbers from previous reports [4, 10, 16, 22]. Generally, involvement of the sigmoid notch was best identified in axial CT reconstructions, as has been noted by other authors [23]. Upon fracture union of the distal radius, underappreciated injuries to the DRUJ are a common cause of ulnar-sided wrist pain [6]. Fractures extending into the radial sigmoid notch may disrupt the DRUJ complex at various levels, thereby altering biomechanics and kinematics of wrist and forearm motion [16, 24]. Since motion in the DRUJ combines translational and rotational components, unappreciated injuries may therefore result in restricted pro- and supination [6, 25].

With the use of computed tomography, the severity of the assessed fractures increased significantly (see Table 1), e.g. in plain X-rays only 14 fractures were classified as multi-fragmentary, intra-articular C3 fractures, whereas CT scans revealed radiocarpal articular surface destruction in 44 cases. This has implications on the decision process of treating this injuries with regard to preventing post-traumatic arthritis. Knirk and Jupiter described already in 1986 [26] that post-traumatic arthritis was seen in patients with an incongruity of the articular surface with a step-off of two millimeters or more. They concluded that achieving and maintaining congruity of the articular surface of the distal part of the radius is paramount for impeding post-traumatic arthritis. In another study about arthritis-predicting factors in distal intraarticular radial fractures, Lutz et al. [27] showed that an increased articular cavity depth should be avoided to prevent degenerative arthritis.

Considering the underestimation of radiocarpal and distal radioulnar joint involvement seen on plain radiographs, the presented results also signify the generous indication for CT scanning in fractures, where conservative treatment is initially anticipated. More severe or rather complex fracture types are to be expected in light of the presented results. Some studies showed that in elderly patients these findings seem to have no influence on wrist function [28, 29], but the clinical relevance in regards to facilitating the decision process of conservative versus surgical treatment especially in younger patients remains to be investigated.

Standard radiographic evaluation demonstrated that fracture involvement of either the DRUJ or the radiocarpal joint rendered fracture extension into the respective other articular compartment very likely. This correlation might be helpful as a rule of thumb in evaluation of DRF in projection radiography.

In a diagnostic study by Bombaci and colleagues [30], the authors obtained standard radiographs and magnetic resonance imaging (MRI) scans in patients with intra-articular DRFs and were able to show that the triangular fibrocartilaginous complex (TFCC) was disrupted in 45% of all cases. With Frykman fracture types VI and VIII the likelihood of a TFCC lesion was significantly higher compared to other fracture subtypes [30]. In the presented analysis, the absolute frequency of Frykman type VI and VIII fractures increased from 23 to 60 cases with the additional information from the CT scan, rendering a TFCC lesion highly likely in a considerable subset of distal radius fracture patients scheduled for ORIF. Although CT scans do not allow evaluation of ligamentous pathologies in the wrist, the complexion of the fracture is more accurately depicted and allows conclusions about concomitant injuries as oppose to standard radiographs.

### Study limitations

As oppose to previous studies [16], intra-articular step-offs and gaps were not quantified within joint surfaces in this study, since the timing of the performed CT scan was not standardized and ranged from immediately post-reduction to fifteen days after injury, with the forearm immobilized in a cast in each case. False “negative” displacement might have been assessed immediately after closed reduction, while after a few days, secondary displacement of truly unstable fractures might still have occurred and delineated incongruences of the respective joints. As of today, the implication of non-displaced intra-articular fractures on functional outcomes remains unclear [10]. Although increased rates of post-traumatic wrist osteoarthritis evaluated with standard radiography have been reported following intra-articular distal radius fractures, these radiological findings did not affect functional outcomes nor patient satisfaction [31, 32]. The clinical relevance of intra-articular fracture extensions into the radiocarpal and distal radio-ulnar joint therefore remains to be investigated.

Only imaging data of patients with distal radius fractures who received surgical treatment were included in this study. Radiographic and computer tomography images of patients who were treated conservatively in a cast were not evaluated in regards to classification mismatch. Since DRFs treated conservatively usually represent milder forms of fracture types and rarely involve intra-articular, multi-fragmentary, or comminuted fractures, the applied classification systems might have shown higher agreements between radiographs and computer tomography scans in these cases.

## Conclusion

Standard radiographic assessments do not allow sufficient evaluation of the articular congruity of the radiocarpal and distal radioulnar joints. To ensure the holistic illustration of a fracture’s complexion, a CT scan is indispensable especially in intra-articular fractures as the severity is underestimated with plain X-rays.
